# Supplementation with SCFAs Re-Establishes Microbiota Composition and Attenuates Hyperalgesia and Pain in a Mouse Model of NTG-Induced Migraine

**DOI:** 10.3390/ijms23094847

**Published:** 2022-04-27

**Authors:** Marika Lanza, Alessia Filippone, Giovanna Casili, Letterio Giuffrè, Sarah Adriana Scuderi, Irene Paterniti, Michela Campolo, Salvatore Cuzzocrea, Emanuela Esposito

**Affiliations:** Department of Chemical, Biological, Pharmaceutical and Environmental Sciences, University of Messina, Viale Ferdinando Stagno D’Alcontres, 98166 Messina, Italy; mlanza@unime.it (M.L.); afilippone@unime.it (A.F.); gcasili@unime.it (G.C.); letterio.giuffre@unime.it (L.G.); sarahadriana.scuderi@unime.it (S.A.S.); ipaterniti@unime.it (I.P.); campolom@unime.it (M.C.); salvator@unime.it (S.C.)

**Keywords:** migraine, short-chain fatty acids, microbiota, intestinal permeability, tight junctions

## Abstract

Migraine is a common brain-disorder that affects 15% of the population. Converging evidence shows that migraine is associated with gastrointestinal disorders. However, the mechanisms underlying the interaction between the gut and brain in patients with migraine are not clear. In this study, we evaluated the role of the short-chain fatty acids (SCFAs) as sodium propionate (SP) and sodium butyrate (SB) on microbiota profile and intestinal permeability in a mouse model of migraine induced by nitroglycerine (NTG). The mice were orally administered SB and SP at the dose of 10, 30 and 100 mg/kg, 5 min after NTG intraperitoneal injections. Behavioral tests were used to evaluate migraine-like pain. Histological and molecular analyses were performed on the intestine. The composition of the intestinal microbiota was extracted from frozen fecal samples and sequenced with an Illumina MiSeq System. Our results demonstrated that the SP and SB treatments attenuated hyperalgesia and pain following NTG injection. Moreover, SP and SB reduced histological damage in the intestine and restored intestinal permeability and the intestinal microbiota profile. These results provide corroborating evidence that SB and SP exert a protective effect on central sensitization induced by NTG through a modulation of intestinal microbiota, suggesting the potential application of SCFAs as novel supportive therapies for intestinal disfunction associated with migraine.

## 1. Introduction

Several trillions of commensal microbes live in the human gut and are collectively known as the gut microbiota; these perform several functions and are considered essential for health and survival [1]. The gut microbiota is an essential component in immune and metabolic health, but it also seems to influence the development and diseases of the enteric and central nervous system (CNS) [2]. The relationship between the gastrointestinal (GI) tract and the brain has been a subject of numerous studies for decades. The specific linkage between the GI tract and the CNS has been termed the “gut–brain axis” and consists of bidirectional communication system mediated by hormonal, immunological and neural signals [3,4]. The gut–brain axis coordinates the gut functions and connects the emotional centers of the brain with the peripheral intestinal functions, including enteric reflex, intestinal permeability, immune activation and enteroendocrine signaling [5,6]. Extrinsic factors, such as dietary habit, lifestyle and infection, as well as intrinsic ones, such as genetic background, play an important role for the gut microbiota profile [7]. Alterations in the intestinal microbiota profile, as a result of psychological and physical stress factors, increase the susceptibility of inflammatory disorders [8,9]. Microbes residing in the gut microbiota may release metabolites and molecules that can trigger the activation of the inflammatory cascade in the CNS through the gut–brain axis, contributing to the initiation and/or progression of various disorders, such as migraine [10]. Migraine headache is often associated with one-sided pain; intolerance to light; and peripheral symptoms in GI environment including nausea, vomiting and diarrhea [11,12]. Although the mechanisms underlying migraine headache are still not fully understood, recent studies have suggested that inflammation and neuroimmune modulation in the GI tract could play an important role in the pathogenesis of migraine headache [13]. However, how gut microbiomes contribute to migraine headache is unclear [14]. Although the treatment for migraine includes the use of triptans—in particular, sumatriptan, [15]—there are still no very efficacious and widely applicable drug treatments for migraine management [15]. Recently, several studies have focused on the beneficial effect of short-chain fatty acids (SCFAs) on various disorders, including migraine [11,16,17]. Sodium butyrate (SB) and sodium propionate (SP) belong to natural SCFAs present in the diet and produced in the colon by the bacterial metabolism of dietary fibers [18]. In in vitro and in vivo studies have demonstrated that SB and SP exert anti-inflammatory, antioxidant and neuroprotective effects via the gut–brain axis [17,18,19]. Scientific evidence has revealed that SB and SP promote intestinal homeostasis and suppress intestinal inflammation through the inhibition of histone deacetylases (HDACs), resulting in the hyperacetylation of core histone proteins (H3 and H4) expressed by some inflammatory-related genes [20]. Moreover, SB and SP are able to inhibit the nuclear factor kappa-light-chain-enhancer of activated B cells’ (NF-κB) translocation, decreasing inflammatory cascade activation [16,18,19]. The anti-inflammatory effects of SB and SP on migraine disorder, as well as their abilities to reduce intestinal inflammation associated with migraine, have been previously well discussed [18]. Therefore, considering the relationship between migraine and GI alterations, this study aimed to investigate the beneficial effects of SB and SP on intestinal permeability and microbiota profile in a mouse model of nitroglycerine (NTG)-induced migraine.

## 2. Results

### 2.1. SCFAs Treatments Attenuates Hyperalgesia and Pain NTG-Induced

**Figure 1:**
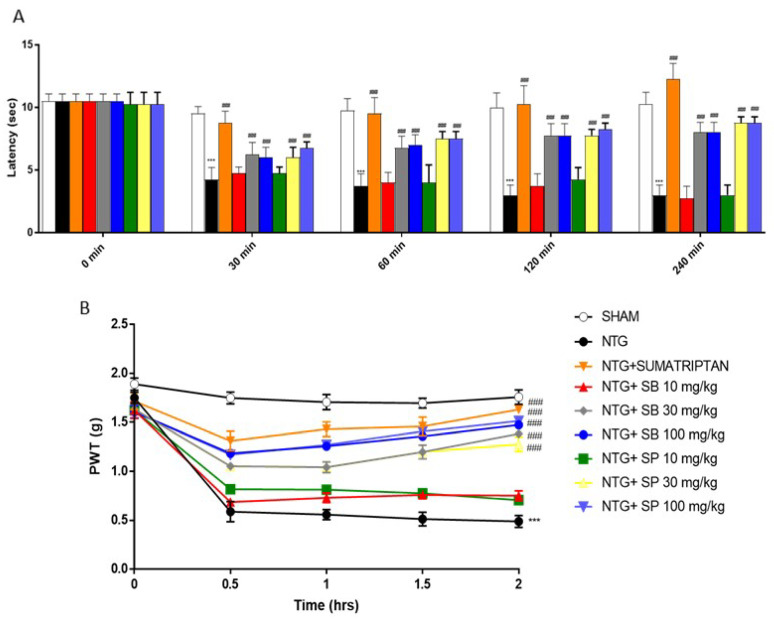
SCFAs treatment attenuates hyperalgesia and pain. SCFAs treatment at the higher doses of 30 and 100 mg/kg significantly increased thermal pain sensation compared to NTG group (**A**). Additionally, the von Frey test revealed that SCFAs treatments at higher doses significantly increased paw-withdrawal thresholds compared to the NTG group (**B**); *** *p* < 0.001 vs. sham, and ### *p* < 0.001 vs. NTG.

Since NTG-evoked hyperalgesia in mice has been developed as a model for sensory hypersensitivity associated with migraine [21], we evaluated the effect of SCFAs on NTG-induced thermal pain sensation by performing the Hargreaves test. First, we confirmed that Sumatriptan treatment, which was used as a negative control, increased the latency time to counteract NTG-induced pain (Figure 1A); however, both SP and SB treatment at the higher doses of 30 and 100 mg/kg significantly increased the latency time to pain reaction, as reported by an increase in latency time up to 240 min after NTG injection (Figure 1A). Moreover, the mechanical allodynia test reported that NTG injection significantly reduced paw-withdrawal thresholds compared to the sham group, and that both SCFAs treatments at higher doses significantly increased that NTG-induced pain (Figure 1B).

### 2.2. SCFAs Modulates Mast Cells Degranulation after NTG-Injection

**Figure 2:**
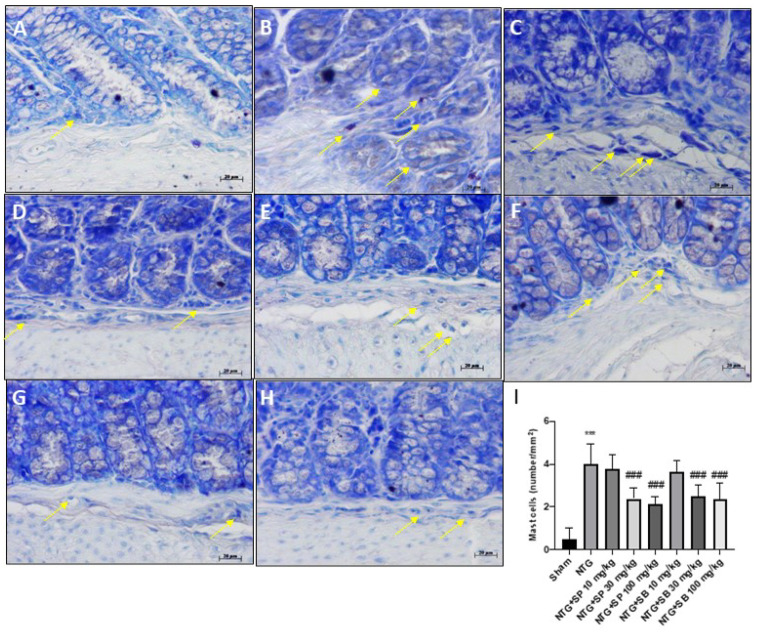
SCFAs treatment reduced mast cell content. Blue toluidine staining revealed that NTG group was characterized by high mast cell content (**B**) compared to sham group (**A**), however the treatment with SCFAs at higher doses significantly reduced mast cell infiltration (**D**,**E**,**G**,**H**). No significant difference was revealed in mice treated with 10 mg/kg of SCFAs (**C**,**F**). The yellow arrows indicated positive cells. (**I**) *** *p* < 0.001 vs. sham; ### *p* < 0.001 vs. NTG.

To evaluate the anti-inflammatory effect of SCFAs in the intestine following NTG injection, we checked the mast cells activation and degranulation on ileum sections. Our results reported that NTG-treated mice were characterized by high levels of in situ mast cells (Figure 2B) compared to the sham group (Figure 2A), whereas the treatment with both SCFAs at higher doses significantly reduced mast cell infiltration (Figure 2D,E,G,H respectively), despite no significant differences found in mice treated with 10 mg/kg of SCFAs (Figure 2C,F, respectively).

### 2.3. Protective Effect of SCFAs on ICAM and P-Selectin Expression

**Figure 3:**
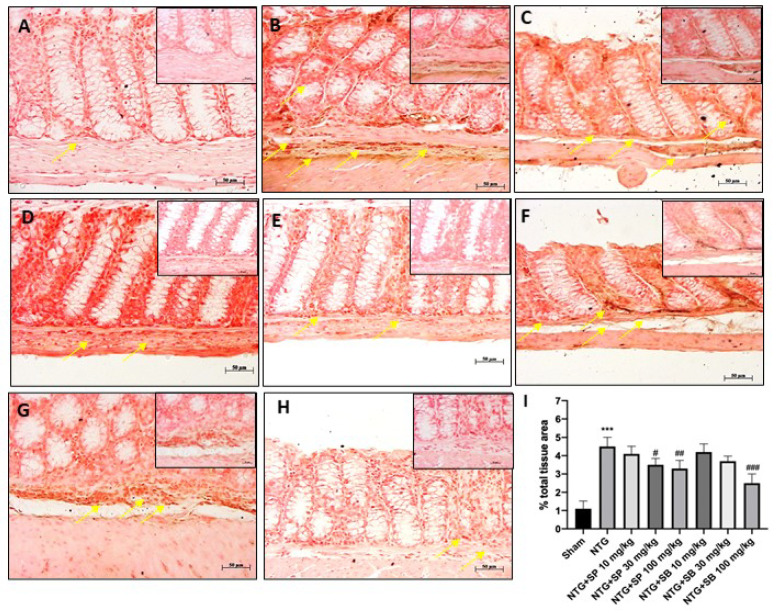
SCFAs treatment decreased ICAM expression. Immunohistochemical localization revealed that NTG group (**B**) was characterized by high expression of ICAM compared to sham group (**A**) however, SCFA treatment (**D**,**E**,**G**,**H**) significantly decreased ICAM expression. No significant difference was revealed in mice treated with 10 mg/kg of SCFAs (**C**,**F**). The yellow arrows indicated positive cells. (**I**) *** *p* < 0.001 vs. sham; # *p* < 0.05 vs. NTG; ## *p* < 0.01 vs. NTG; ### *p* < 0.001 vs. NTG.

**Figure 4:**
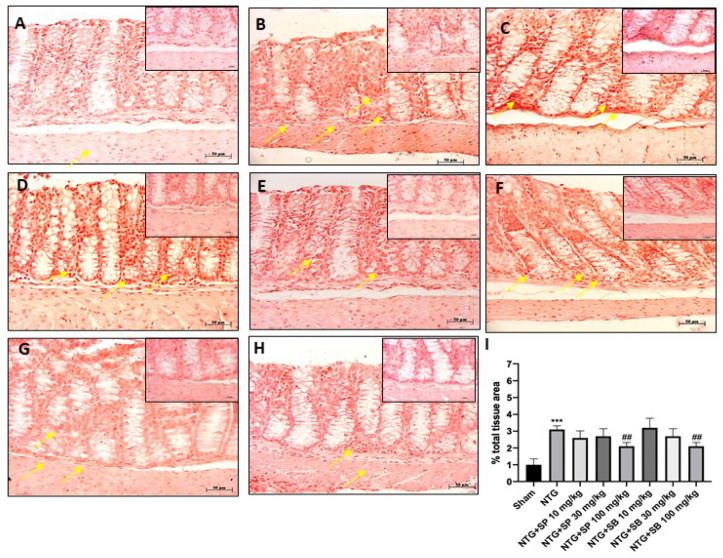
SCFAs treatment reduced P-selectin expression. Immunohistochemical localization revealed that NTG group (**B**) was characterized by high expression of P-selectin compared to sham group (**A**) however, SCFA treatment (**D**,**E**,**G**,**H**) significantly decreased P-selectin expression. No significant difference was revealed in mice treated with 10 mg/kg of SCFAs (**C**,**F**). The yellow arrows indicated positive cells. (**I**) *** *p* < 0.001 vs. sham; ## *p* < 0.01 vs. NTG.

Scientific evidence has demonstrated the key role of intestinal mucosal integrity in the pathogenesis of inflammatory diseases [22]. Therefore, in this study, we investigated the effect of SCFAs on intestinal mucosal integrity by evaluating ICAM and P-selectin expression on ileum. Our results demonstrated that NTG group was characterized by a high percentage of labeled epithelial cells for ICAM and P-selectin compared to the sham group (Figure 3A,B and Figure 4A,B, respectively); however, SCFAs administration at a higher dose to NTG-injected mice significantly reduced their expression (Figure 3E,H and Figure 4E,H, respectively); there was no significant difference with the NTG group for mice treated with 10 mg/kg of SCFAs (Figure 3C,F and Figure 4C,F, respectively).

### 2.4. Effect of SCFAs on Tight Junctions (TJs) Expression

**Figure 5:**
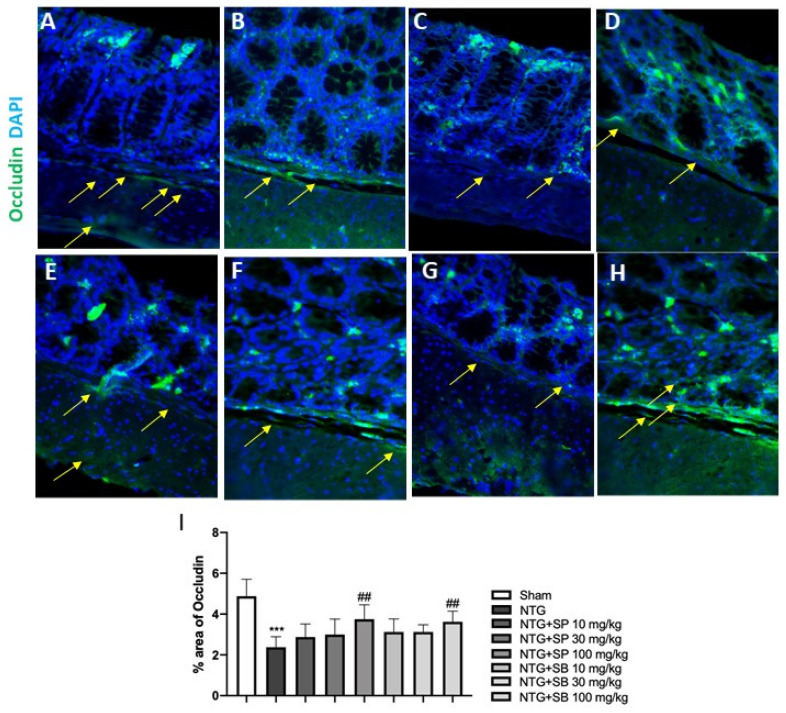
SCFAs treatment restores occludin expression. Immunofluorescence staining revealed that NTG group was characterized by a decrease of occludin expression (**B**) compared to sham group (**A**), however SCFAs treatment (**E**,**H**) significantly restored occludin expression. No significant difference was observed in SP- and SB-treated-mice at the lower dose of 10 mg/kg (**C,D,F**,**G**). The yellow arrows indicated positive cells. (**I**) *** *p* < 0.001 vs. sham; ## *p* < 0.01 vs. NTG.

**Figure 6:**
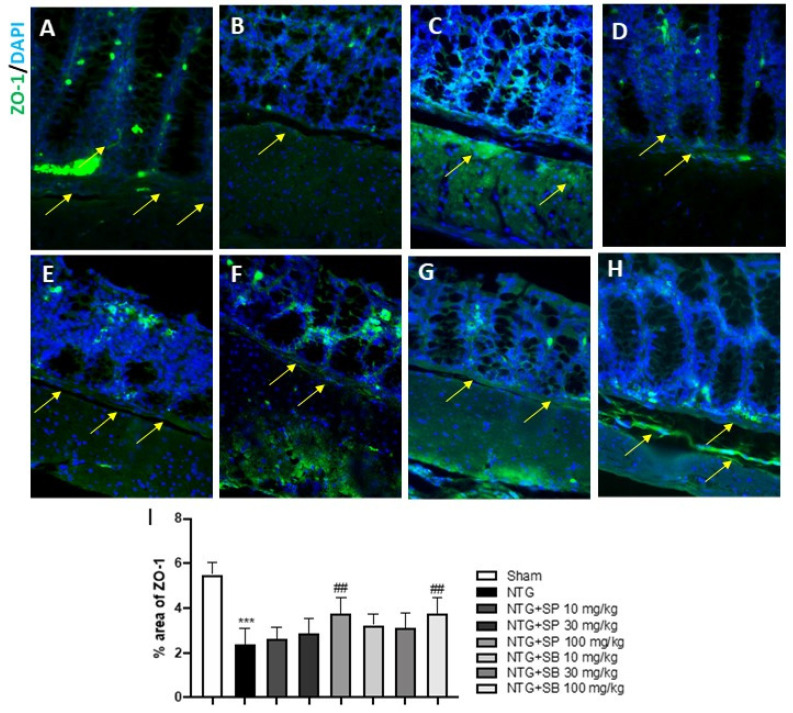
SCFAs treatment restores ZO-1 expression. Immunofluorescence staining revealed that NTG group was characterized by a decrease of ZO-1 expression (**B**) compared to sham group (**A**), however SCFAs treatment (**E**,**H**) significantly restored ZO-1 expression. No significant difference was observed in SP- and SB-treated-mice at the lower dose of 10 mg/kg (**C,D,F**,**G**). The yellow arrows indicated positive cells. (**I**) *** *p* < 0.001 vs. sham; ## *p* < 0.01 vs. NTG.

It has been proven that migraine may result from intestinal permeability alteration [23]. Intestinal-barrier function is guaranteed by the presence of TJs, in particular, occludin and ZO-1, which play a key role in the maintenance of gut permeability [24]. Thus, in this study, we decided to investigate the effect of SCFAs on TJs’ expression, following NTG-injection, on ileum section by immunofluorescence staining (IF). Our results showed that NTG injection provoked a significant decrease of occludin and ZO-1 expression compared to the sham group (Figure 5A,B and Figure 6A,B, respectively); meanwhile, the treatment with both SP and SB at higher doses significantly restored occludin and ZO-1 expression (Figure 5E,H and Figure 6E,H, respectively); no significant difference was observed in SP- and SB-treated-mice at the lower dose of 10 mg/kg (Figure 5C,D,F,G and Figure 6C,D,F,G).

### 2.5. Effect of SCFAs on Intestinal Permeability

**Figure 7:**
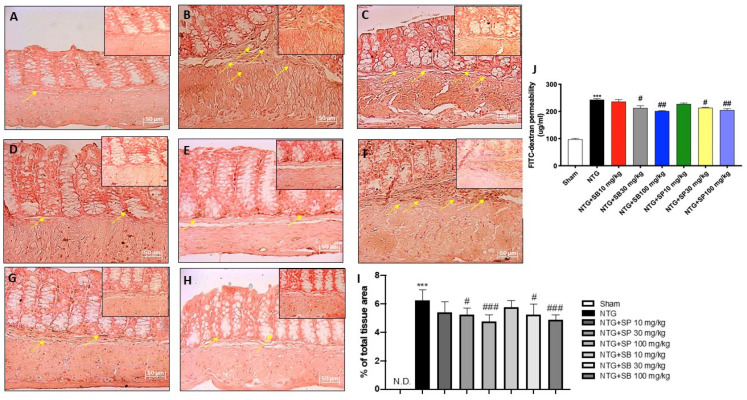
SCFAs treatment re-establishes intestinal permeability. Immunohistochemical localization revealed that NTG group (**B**) was characterized by high expression of E-cadherin compared to sham group (**A**) however, SCFA treatment (**D**,**E**,**G**,**H**) significantly decreased E-cadherin expression. No significant difference was revealed in mice treated with 10 mg/kg of SCFAs (**C**,**F**). The yellow arrows indicated positive cells. N.D. = not detected. FITC–Dextran assay revealed that SCFAs treatment significantly inhibited the increase in intestinal permeability (**J**). (**I**) *** *p* < 0.001 vs. sham; # *p* < 0.05 vs. NTG; ### *p* < 0.001 vs. NTG. (**J**) *** *p* < 0.001 vs. sham; # *p* < 0.05 vs. NTG; ## *p* < 0.01 vs. NTG.

An altered intestinal homeostasis and permeability has been linked to an increased expression of E-cadherin, which is essential for maintaining the cell–cell contact and regulating cytoskeletal complexes [25]; here, we confirmed that NTG mice showed a significant increase of E-cadherin expression when compared to the sham group (Figure 7A,B, respectively); meanwhile, SCFAs treatments at the higher doses (30 and 100 mg/kg) significantly reduced the E-cadherin expression (Figure 7D,E,G,H, respectively); no significant differences found in mice treated with 10 mg/kg of SCFAs (Figure 7C,F). Moreover, the intestinal permeability was evaluated using FITC-Dextran assay. The intestinal permeability was significantly increased in the NTG-group compared to sham group, however SCFAs treatment at higher doses significantly inhibited the increase in intestinal permeability as shown in the Figure 7J; despite no significant difference found in mice treated with 10 mg/kg of SCFAs.

### 2.6. Effect of SCFAs on Microbiota Composition

**Figure 8:**
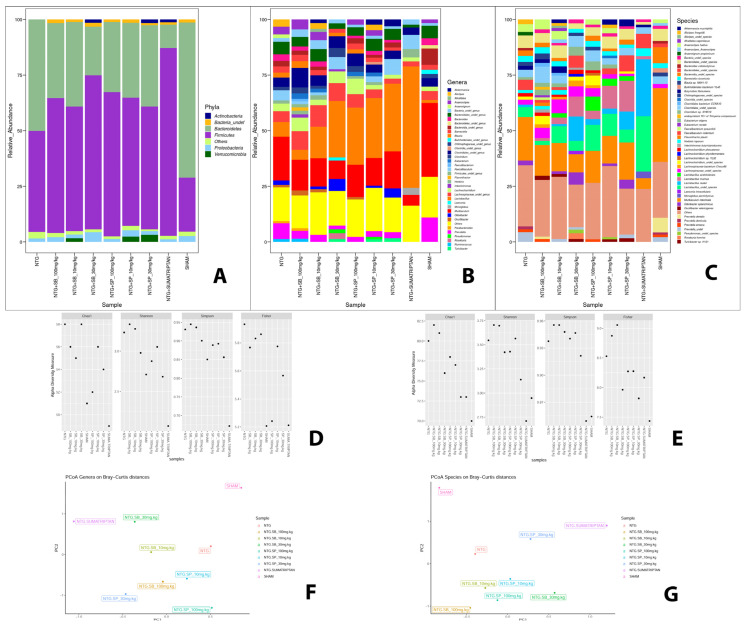
Overall intestinal microbiota composition and diversity over NTG-induced migraine, treatment with SP, SB and sumatriptan. Most abundant (**A**) phyla, (**B**) genera and (**C**) species observed in this study among the different conditions. Content in species explained by alpha diversity at genus (**D**) and species (**E**) levels. PCO plots based on Bray–Curtis, distance showing the differences among the intestinal microbial community of our sample at genus (**F**) and species (**G**) level.

A total of 969,152 raw reads were sequenced by Illumina MiSeq and merged in 862,263 fragments (~89.6%), of which over 99.6% showed a Phred-score >20 (Table 1). After taxonomic assignment, 818,187 paired reads found a hit in the SILVA database, leading to the identification of 11 phyla, 80 genera and 126 species with a minimum relative abundance ≥ 0.1%. Overall, the most abundant phylum was *Firmicutes* (~57.1%) followed by *Bacteroidetes* (~36.2%), *Proteobacteria* (~2.1%), an undefined phylum (~1.2%) and *Verrucomicrobia* (~1.1%) (Figure 8). However, considering the relative abundance in each group individually, *Firmicutes* was the main phylum in the samples treated with SB, SP and sumatriptan (ranging from ~55.3 to~84.5%), whereas *Bacteroidetes* was the most abundant phylum in NTG and sham group (~50.1% and ~69.9% respectively), followed by *Firmicutes* (~45.4% and ~24.1%, respectively). The statistical analysis showed also that *Firmicutes* was enriched in SB, SP, sumatriptan and migraine groups compared to the sham (*q*-value < 0.25), whereas *Bacteroidetes* was enriched in the sham group (*q*-value < 0.25). At the genus level, the number of genera observed with a relative abundance ≥0.1% was similar among all the groups tested, ranging from 58 genera for SB 30 mg/kg and NTG to 49 for sumatriptan, for a total of 80 different genera, and 32 of those were shared by all samples included in this study. Considering the relative abundance of each group, we observed some differences such as those obtained at phylum level. Notably, *Lactobacillus* was the most abundant genus in the groups treated with SP, SB and sumatriptan, except for SB 100 mg/kg, and was statistically associated with groups treated with SP and sumatriptan. Moreover, statistical analysis showed that the genus *Bifidobacterium* was associated with the SP, SB and sumatriptan groups. Otherwise, the NTG and sham groups showed a higher proportion of two genera, *Bacteroidetes* and *Muribaculum*, which were the main genera in both groups, and *Prevotella (q*-value < 0.25). Moreover, the NTG group with induced migraine showed a higher proportion of some genera, e.g., *Alistipes*, compared to a different dose of treatment, and in the same way, different undefined genera, such as *Chitinophagaceae* undefined genus and *Burkholderiales* undefined genus, showed a higher relative abundance in the sham group (*q*-value < 0.25). Notably, among the SP and SB groups, several genera identified in this study appear more abundant proportionally to the dose of these molecules. In fact, the genera *Faecalibacterium*, *Turicibacter* and *Odoribacter* showed a higher relative abundance in the sample treated with 30 mg/kg of SB and SP, and 10 mg/kg, while *Cytophaga* showed an increased proportion in the groups treated with 100 mg/kg of SB and SP (Figure 8). Among the 126 species identified, 39 were shared among all samples, with a relative abundance ≥0.1%. No significance differences were found in terms of number of species observed among the groups that ranged from 82 (SB 100 mg/kg) to 70 (sumatriptan). *Muribaculum intestinale* was the most abundant species in the sham group, NTG group and groups treated with SP and SB, expect for SB 30 mg/kg, while the sumatriptan group showed a particularly lower proportion. However, based on its relative abundance, this species was statistically associated with the sham (~33.2%) and NTG (~19.6%), but no evidence of an association was found with the treated groups. Notably, groups treated with sumatriptan, SB and SP showed a higher proportion of *Lactobacillus* spp. than the sham and NTG groups. In fact, the species *Lactobacillus reuteri*, *Lactobacillus murinis*, *Lactobacillus acetotollerans, Lactobacillus delbrueckii, Lactobacillus helveticus, Lactobacillus fermentum, Lactobacillus jensenii, Lactobacillus paracasei, Lactobacillus plantarum, Lactobacillus paraplantarum* and an undefined species of *Lactobacillus* were found at higher proportions in sumatriptan and in all SB and SP groups (*q*-value < 0.25). Among those, the sumatriptan group showed a notably higher proportion of *Lactobacillus* undefined and *Lactobacillus reuteri* (~24.8% and ~25.6%, respectively) compared to that of the SP and SB groups (ranging from ~11.1% to ~2% and from ~11% to ~0.6%). Among the groups treated with SP and SB at different doses, finally, the species *Prevotella dentalis* was considerably enriched in the microbiota of the sham group (~6.5%) in relation to the other conditions tested in this study (*q*-value < 0.25) (Figure 8).

## 3. Discussion

Migraine is a complex and multifactorial brain inflammatory disease [12]. Several studies suggest that migraine could be associated with GI disorders, including inflammatory bowel disease [26]. It has been demonstrated that migraine is related to an increased intestinal permeability, a compromised gut-barrier function and microbiota profile alteration [7,26]. The gut microbiota’s main function is to regulate the absorption of nutrients and preserve intestinal integrity and permeability [27]. The gut microbiota also plays a key role in the bidirectional communication between the gut and brain, suggesting that the gut microbes may shape neural development, modulate neurotransmission and affect behavior, contributing to the pathogenesis and/or progression of many diseases, including migraine [28,29]. Furthermore, much evidence supports the link between altered intestinal permeability and neurological conditions such as migraine [29,30]. The intestinal mucosa act as a semi-permeable barrier which regulates the absorption of nutrients and avoid the passage of substances or pathogens that are potentially harmful in the intestinal lumen [27,31]. Intestinal permeability is guaranteed by the presence of TJs, multiprotein complexes which regulate ion, water and solute diffusion to maintain gut homeostasis and barrier function [27]. However, this balance can be altered in response to inflammatory stimuli and neurological conditions.

In the last decade, several studies have focused on the effects of SCFAs in various inflammatory brain disorders, including migraine [17,18,19]. SCFAs, produced in the colon by the anaerobic fermentation of undigested carbohydrates, regulate gut permeability and maintain gut homeostasis and barrier function [18,32,33]. Among SCFAs, SB and SP are particularly well-known to exert neuro-protective effects and suppress intestinal inflammation [17,19,28,33]. The anti-inflammatory effects of SB and SP on migraine disorder, as well as their abilities to reduce intestinal inflammation associated to migraine, have been well discussed previously [18,32]. Therefore, considering the relationship between migraine and altered gut permeability, here we evaluated the effects of SB and SP on microbiota composition and intestinal permeability in a mouse model on NTG-induced migraine.

Firstly, we decided to evaluate the effects of SB and SP on symptoms associated with migraine headache by conducting behavioral tests. A common and debilitating symptom of migraine headache is photophobia due to an important activation of the trigeminovascular system (TGVS), reflecting an allodynic response activation to a painful stimulus [21]. Our results demonstrated that both SB and SP treatments at higher doses significantly reduced mechanical allodynia caused by NTG administrations, relieving stimulus-evoked spontaneous nociception.

Although the exact mechanisms are not still clear, scientific evidence suggests that alteration of gut microbiota profile can contribute to the initiation and/or progression of migraine by the release of numerous inflammatory mediators in the intestine and the recruitment of mast cells as important effectors of the gut–brain axis [5,18,34]. Our results clearly demonstrated that NTG injection provokes an evident inflammatory state that is characterized by a high content of mast cells in the intestine; however, both SB and SP treatments at higher doses are able to decrease the number of mast cells and their degranulation, confirming the anti-inflammatory properties.

Furthermore, recent studies have shown that migraine disorder is associated with an intestinal mucosal integrity alteration, followed by the barrier function being compromised [12]. Intestinal-barrier integrity is guaranteed by the presence of epithelial adhesion molecules which regulate gut mucosal homeostasis [35]. However, it has been proven that intestinal inflammation can alter the expression of adhesion molecules, including ICAM and P-selectin, which play a critical role in the neutrophil adhesion process to and migration across the mucosal membrane [31]. In this context, our results clearly demonstrated that ICAM and P-selectin expression were significantly decreased in SCFA-treated mice compared to the NTG group. On the other hand, it has been proven that alterations in the gut microbiota have been associated with modifications of gut-barrier function [24]. Intestinal-barrier function is guaranteed by the presence of tight junctions (TJs); in particular, ZO-1 and occludin, localized to the apical–lateral membrane junction, regulate the absorption of nutrients, electrolytes and water [24]. Alterations of TJs formation and distribution and/or destabilization of the TJ complexes lead to intestinal epithelial-barrier dysfunction [24]. According to this evidence, our results showed that NTG injection provokes a significant decrease of occludin and ZO-1 levels; however, both SB and SP treatments at higher doses restore TJs expression almost to basal levels, re-establishing intestinal permeability. Additionally, an altered intestinal homeostasis has been linked to an increased expression of E-cadherin, a major component of adherent junctions in the intestine [25]. In this regard, our results demonstrated that both SB and SP treatments at higher doses significantly reduced E-cadherin expression, suggesting that SB and SP could alleviate the symptoms of migraine associated with intestinal-barrier alteration; additionally, the effects of SB and SP on intestinal permeability are confirmed also by FITC–Dextran assay. Moreover, migraine disorder is characterized by an alteration of gut microbiota composition [36]. Our genetic data revealed that some bacterial taxa could be associated with the conditions tested in this study. *Firmicutes* and *Bacteroidetes*, Gram-negative bacteria, represent an important component of the intestinal microbiota that is involved in various biological functions, such as the absorption of nutrients in the intestinal tract [37]. A higher ratio of *Firmicutes/Bacteroidetes* was found in all groups treated with SP, SB and sumatriptan. The increasing abundance of *Firmicutes* in the intestinal microbiota was already described in several studies regarding the effect of a chronic opioid therapy against several diseases [38]. This finding correlates also with the higher concentration of *Bacteroidetes* and *Muribaculum* spp. in the sham group. *Muribaculum* is a genus that was discovered in 2016 [37,39], and although it represents one of the most important genus components of the intestinal microbiota of mice, its role inside this bacterial community is still unclear. Until now few studies have reported that *Muribaculum intestinale* could be associated with a healthy intestinal microbiota in mice and the blind mole-rat *Spalax leucudon*, [40,41,42]. Moreover, in this study, we describe an enrichment of several species belonging to the *Lactobacillus* genus in a group treated with SP, SB and sumatriptan. On the contrary, *Lactobacillus* spp. represents one of the most important and well-studied bacterial genera found as a normal component of intestinal microbiota of several mammalian species, wherein it is involved in several beneficial function, such as the suppression of pathogens, immunomodulation, stimulation of epithelial cell proliferation and differentiation, fortification of the intestinal barrier and restoration of homeostasis in intestinal disorders; thus, it plays a protective role against inflammatory diseases [42]. Different studies have shown that the oral administration of probiotics containing different bacterial strains, including several *Lactobacillus* species, could relieve or reduce the duration and frequency of migraine attack, thus improving the quality of life [43]. Moreover, it was also demonstrated that the administration of several *Lactobacillus* strains could lead to an improvement in the absorption of SCFA by the gastroenteric epithelial cells [33,44]. In accordance with this evidence, the increased abundance of *Lactobacillus* spp. found in SP, SB and sumatriptan groups, reducing the general inflammatory degree, may have helped decrease migraine attack in the tested animals.

## 4. Materials and Methods

### 4.1. Animals

CD1 mice (females, 25 to 30 g, Envigo, Italy) were housed in a controlled environment (22 ± 2 °C, 55 ± 15% relative humidity, 12 h light/dark cycle). Animals were fed with a standard diet and water ad libitum. The study was approved by the University of Messina Review Board for the care of animals. Animal care was in compliance with Italian regulations on protection of animals used for experimental and other scientific purposes (Ministerial Decree 16192) as well as with the Council Regulation (EEC) (Official Journal of the European Union L 358/112/18/1986). Authorization n° 368/2019-PR released in 14 May 2019.

### 4.2. NTG-Migraine Model

NTG was prepared from a stock solution of 5.0 mg/mL nitroglycerin in 30% alcohol, 30% propylene glycol and water (American Reagent Inc. 5 Ramsey Road Shirley, NY, USA 11967). NTG was freshly diluted in 0.9% saline to a final dose of 10 mg/kg. The vehicle used in these experiments for the sumatriptan-, SB- and SP-treated groups was 0.9% saline [21]. Animals were treated orally with SP at the doses of 10, 30 and 100 mg/kg, and then SB at the doses of 10, 30 and 100 mg/kg 5 min following NTG injection. For the sumatriptan group, animals received sumatriptan orally, at the dose of 600 μg/kg, 5 min after NTG injection. Mice were sacrificed 4 h following NTG injection, and their intestinal tissue was removed to perform the analyses.

#### Experimental Groups

Mice were randomly divided into the following groups:

− Group sham + vehicle (veh): mice received saline;
− Group NTG: mice received NTG (10 mg/kg) intraperitoneally (i.p.);
− Group NTG+ sumatriptan: mice received sumatriptan orally (600 μg/kg) 5 min after NTG (10 mg/kg) (i.p.);
− Group NTG+ SP 10 mg/kg: mice received SP orally at a dose of 10 mg/kg 5 min after NTG injection (i.p.);
− Group NTG+ SP 30 mg/kg: mice received SP orally at a dose of 30 mg/kg 5 min after NTG injection (i.p.);
− Group NTG+ SP 100 mg/kg: mice received SP orally at a dose of 100 mg/kg 5 min after NTG injection (i.p.);
− Group NTG+ SB 10 mg/kg: mice received SB orally at a dose of 10 mg/kg 5 min after NTG injection (i.p.);
− Group NTG+ SB 30 mg/kg: mice received SB orally at a dose of 30 mg/kg 5 min after NTG injection (i.p.);
− Group NTG+ SB 100 mg/kg: mice received SB orally at a dose of 100 mg/kg 5 min after NTG injection (i.p.).

The minimum number of mice for every technique was estimated with the statistical test “ANOVA: Fixed effect, omnibus one-way”, with G-power software. This statistical test generated a sample size equal to N = 8 mice for each technique.

Data regarding the groups of control mice (sham+ SP 10 mg/kg, sham+ SP 30 mg/kg, sham+ SP 100 mg/kg, group sham+ SB 10 mg/kg, sham+ SB 30 mg/kg and sham+ SB 100 mg/kg) are not shown, because SP and SB alone demonstrated no significant histological changes compared to the sham +veh group. The doses of SP and SB were based on a previous dose–response study in our laboratory [18]. The dose of sumatriptan was chosen according to the respective literature [45]. The sumatriptan group was subjected to behavioral tests to assess migraine and perform a microbiota composition analysis.

Previously, the authors tested the control group with the NTG vehicle alone (30% alcohol, 30% propylene glycol and water) in another study [21]; our previous results demonstrated that the vehicle (30% alcohol, 30% propylene glycol and water) did not show any toxic effect; therefore, the authors decided to indicate the control group with only saline.

### 4.3. Behavioral Tests

#### 4.3.1. Hargreaves Test

To determine the thermal nociceptive thresholds, we used the Hargreaves assay, which focuses radiant light on the hind paw and measures the latency in seconds to withdrawal of the hind paw (PAW Thermal Stimulator, UC San Diego Department of Anesthesia, San Diego, CA, USA). For each animal, the withdrawal latency is the average of three separate determinations, taken with at least 2 min between each trial. Thermal nociceptive was measured immediately before and 30, 60, 120 and 240 min after injection of NTG [46].

#### 4.3.2. Von Frey Test

The von Frey test is a method used to evaluate mechanical allodynia in rodents. For the application of von Frey filaments, mice are placed one by one on a small elevated platform, and a monofilament is applied perpendicularly to the plantar surface of the hind paw until it bends. A positive response is brisk paw withdrawal, licking or shaking of the paw during the application of the monofilament or immediately after the removal of the filament, as previously described [47].

### 4.4. Blue Toluidine Staining

Ileum sections were stained with toluidine blue (Bio-Optica, Milano, Italy) to evaluate mast cell amount and their degranulation [48]. The number of metachromatic stained mast cells was obtained by counting five high-power fields for the section, using an Axiovision Zeiss (Milan, Italy) microscope and the correlated AxioVision software (Carl Zeiss Vision, Jena, Germany). Data were reported as the mean with standard deviation (SD). Images are shown at 40× magnification (20 µm scale bar).

### 4.5. Immunohistochemical Localization of ICAM, P-Selectin and E-Cadherin

Immunohistochemical localization was performed on ileum sections, as previously described [18]. Ileum sections were incubated at room temperature, overnight (O/N), with the following primary antibodies: E-cadherin (1:100, Santa Cruz Biotechnology, Dallas, TX, USA, sc-21791), intercellular adhesion molecule (ICAM) (1:100, Santa Cruz Biotechnology, Dallas, TX, USA, sc-7891) and P-selectin (1:100, Santa Cruz Biotechnology, Dallas, TX, USA, sc-6941). At the end of the incubation with the primary antibodies, the sections were washed with PBS and incubated with a secondary antibody (Santa Cruz Biotechnology, Dallas, TX, USA) for 1 h at 37 °C. The reaction was revealed by a chromogenic substrate (brown DAB) and counterstaining with NUCLEAR FAST-RED. For immunohistochemistry, 20× (50 µm scale bar) and 40× (20 µm scale bar) were shown.

### 4.6. Immunofluorescence Analysis of Occludin and ZO-1

Immunofluorescence assay was performed on ileum sections, as previously described [18]. Ileum sections were incubated with the following primary antibodies: anti-zonula occludens-1 (ZO-1) (617300 Invitrogen, Carlsbad, CA, USA, 1:100 in PBS, *v*/*v*) and anti-occludin (71–1500 Invitrogen, Carlsbad, CA, USA; 1:100 in PBS, *v*/*v*) at 37 °C, overnight. Then tissue sections were washed with PBS and incubated with secondary antibody anti-mouse Alexa Fluor-488 antibody (1:1000 *v*/*v*, Molecular Probes, Altrincham, UK) for 1 h at 37 °C. For nuclear staining, 4′,6′-diamidino-2-phenylindole (DAPI; Hoechst, Frankfurt, Germany) (2 μg/mL) in PBS was added. Sections were observed and photographed at 40× magnification, using a Leica DM2000 microscope.

### 4.7. Intestinal Permeability Measurement

Intestinal permeability measurements after NTG-migraine induction on animals was performed by using 4 kDa of fluorescein isothiocyanate-conjugated (FITC) dextran, as previously described by Woting et al. [49].

### 4.8. DNA Extraction from Tools

Total microbial DNA was extracted by a commercial DNA extraction kit (Nucleospin Tissue Macherey-Nagel, Düren, Germany), according to the manufacture’s protocols. Quality and integrity of total microbial DNA were evaluated, respectively, spectrophotometrically by Nanodrop, and by the visualization of 1 µL of sample on 1.3% electrophoresis agarose gel. High-quality DNA (A260/A280 ≥ 1.8; A260/A230 ≥ 1.8) was selected for the amplification and V3–V5 hypervariable regions of the bacterial universal gene coding for the 16S rRNA, and PCR products were used for library preparation. Sequencing was performed in paired end (2 × 300), using Illumina MiSeq (Illumina, San Diego, CA, USA) at Eurofins Genomics (Ebersberg, Germany).

### 4.9. Bioinformatics Pipeline

A first quality check of raw reads was performed by using FastqC (www.bioinformatics.babraham.ac.uk/projects/fastqc, version 0.11.9 (accessed on 17 June 2021)). Then adapters and low-quality reads (Phred-score < 20) were removed, using Trimmomatic V. 0.39 [50]. Cleaned reads that showed an overlapping at least of 10 bp were merged by using PEAR (version 0.9.11) [51], and taxonomic assignment was performed by using Kraken2 [52] (version 2.12) against the SILVA database (version 132). Relative abundances were calculated at phylum, genus and species level for each sample, and then all taxa that showed a relative abundance < 0.1% were filtered out. Alpha diversity indexes and all plots were generated by using the packages vegan (version 2.5.7) and ggplot2 (version 3.3.5) integrate in R! (Version 4.1.2) [53]. Based on the relative abundances, statistical associations between taxa and the different treatments considered in this study were explored with Maaslin2 [54]. A list containing the principal commands used for the microbiota characterization with relative description is provided in the Supplementary Materials, which are available online.

### 4.10. Materials

All compounds and other chemicals were obtained from Sigma-Aldrich (Milan, Italy). All stock solutions were prepared in non-pyrogenic saline (0.9% NaCl; Baxter, Milan, Italy).

### 4.11. Statistical Evaluation

All values are indicated as the mean ± standard error of the standard deviation (SD) of N observations. The experiment is descriptive, as a minimum of three experiments were performed on different days on tissue sections collected from all animals in each experimental group. Data were analyzed with the GraphPad Prism software 7.04 and by one-way ANOVA, followed by a Bonferroni post hoc test for multiple comparisons. A *p*-value of less than 0.05 was considered significant.

## 5. Conclusions

In conclusion, the obtained results offer new insight into the role of SCFAs in migraine pathogenesis, as well as on microbiota profile and intestinal permeability. The data demonstrated that SP and SB treatment significantly restored intestinal permeability and integrity and re-established the microbiota composition following NTG-induced migraine. Therefore, based on these findings, SB and SP could be used as a future strategy to promote healthy gut microbiota when brain disorders such as migraines occur.

## Figures and Tables

**Table 1:** Bioinformatic metrics and number of Taxa identified. Marked with dash the number of unique taxa found through the samples.

	V3–V5						
	Raw Reads	Merged	Clean Reads	Classified	Phylum	Genera	Species
Sham	111,002	97,172	96,915	90,264	8	51	70
NTG+ SB 100 mg/kg	108,808	98,049	97,731	92,342	6	56	82
NTG+ SB 10 mg/kg	132,836	119,373	118,943	114,293	8	55	81
NTG+ SB 30 mg/kg	83,828	75,125	74,849	71,517	8	58	76
NTG+ SP 100 mg/kg	122,776	109,779	109,443	104,130	8	52	78
NTG+ SP 10 mg/kg	106,006	93,551	93,251	89,616	8	56	77
NTG+ SP 30 mg/kg	110,765	97,279	96,941	92,845	9	54	73
NTG	119,745	107,072	106,740	101,596	8	58	80
NTG+ Sumatriptan	73,386	64,863	64,635	184,014	8	49	73
Total	969,152	862,263	859,448	940,617	11 *	80 *	126 *

## Data Availability

The authors declare that all data and materials supporting the findings of this study are available within the article. The data that support the findings of this study are available from the corresponding author upon reasonable request.

## Supplementary Materials

The following supporting information can be downloaded at: https://www.mdpi.com/article/10.3390/ijms23094847/s1.
